# Colovaginal anastomosis: an unusual complication of stapler use in restorative procedure after Hartmann operation

**DOI:** 10.1186/1477-7819-3-74

**Published:** 2005-11-15

**Authors:** Zhongshu Yan, Guoqing Liao

**Affiliations:** 1 Department of Gastrointestinal Surgery, Xiangya Hospital, Central South University, Changsha 410008, China

## Abstract

**Background:**

Rectovaginal fistula is uncommon after lower anterior resection for rectal cancer. The most leading cause of this complication is involvement of the posterior wall of the vagina into the staple line when firing the circular stapler.

**Case presentation:**

A 50-year-old women underwent resection for obstructed carcinoma of the sigmoid colon with Hartmann procedure. Four months later she underwent restorative surgery with circular stapler. Following which she developed rectovaginal fistula. A transvaginal repair was performed but stool passing from vagina not per rectum. Laporotomy revealed colovaginal anastomosis, which was corrected accordingly. Patient had an uneventful recovery.

**Conclusion:**

Inadvertent formation of colovaginal anastomosis associated with a rectovaginal fistula is a rare complication caused by the operator's error. The present case again highlights the importance of ensuring that the posterior wall of vagina is away from the staple line.

## Background

Rectovaginal fistula is uncommon after lower anterior resection for rectal cancer. The most leading cause of this complication is involvement of the posterior wall of the vagina into the staple line when firing the circular stapler. Here we report a very rare case developed in a restorative surgery after Hartmann procedure for cancer of sigmoid colon.

## Case presentation

A 50-year-old woman was admitted to a county hospital in the suburb of Shanghai in August 2003 with acute colonic obstruction due to cancer of the sigmoid colon confirmed by colonoscopy. She underwent an emergency surgical resection of the tumor followed by end sigmoid colostomy and closure of the rectal stump at 10 cm above the anal verge (Hartmann procedure). The pathology was reported as well differentiated adenocarcinoma of the sigmoid without lymph node metastasis. Her postoperative course was uneventful. Asking for a restorative surgery four months later she was readmitted to the same hospital and operated by the same surgeon. There was no much adhesion in the pelvis and an end-to-end anastomosis was made using a circular stapler (Proximate^® ^29 mm, Ethicon). She was discharged from the hospital after three weeks. After her return to home she noticed passage of gas and later feces from vagina. She visited the surgeon's clinic again and had a check-up by the gynecologist. The gynecologist did not find her cervix uteri but a defect was found on her posterior vaginal wall five centimeter above the vaginal orifice with feces coming out. The surgeon denied any possibility of removal of her uterus during the surgery. At that point no treatment for rectovaginal fistula was given to the patient. Her case was presented to the medical malpractice committee for evaluation and she came to us to seek further treatment five months after her second operation.

Digital examination of the rectum revealed a 1.5 cm defect on the anterior anal wall 5 centimeters above the anal verge. Vaginal inspection showed three metal clips stapled both the anterior and posterior vaginal wall with small cleavages between the clips. Stool discharged from the vagina. The cervix uteri was not found. Magnetic resonance imaging (MRI) showed that the uterus was present closed to the sacrum. Before this admission she had had a colonoscopy undertaken in a university hospital in Shanghai which reported that the whole colon and the anastomotic site were otherwise normal but a fistula on the anterior rectal wall. The diagnosis of rectovaginal fistula was established and she was operated under epidural anesthesia. Transvaginal repair of rectovaginal fistula was performed. The patient was uneventful until the sixth postoperative day when she passed flatus and stool per vagina but not the rectum. Failure of repair was highly suspected and she was taken into the operation room and had an examination under anesthesia. A blind rectal remnant was found about ten centimeters above the anal orifice and the repaired rectovaginal fistula was all right. Vaginal examination found that after removal of the residual metal clips there was an opening at the anterior fornix of vagina which was three centimeters above the original rectovaginal fistula. The diagnosis of an inadvertent colovaginal anastomosis was established then and a laparotomy was undertaken. After dissection of dense adhesion in the low abdominal cavity and pelvis the uterus was found to be adhered to the sacropromotory and the sigmoid colon was located anteriorly across the left appendix uteri and ended in the anterior cul-de-sac. There was dense adhesion in this area indicating that there has been dissected during the previous surgery. The colovaginal anastomosis was identified by insertion of finger within the vagina. After detachment of the colovaginal anastomosis and repair of the anterior wall of vagina the sigmoid colon was anastomosed to the rectal remnant by a circular stapler (Proximate^® ^29 mm, Ethicon). Postoperative period was uneventful and the patient was discharged from the hospital eight days after the final operation. She was followed up nine months later with satisfactory result.

## Discussion

**Figure 1:**
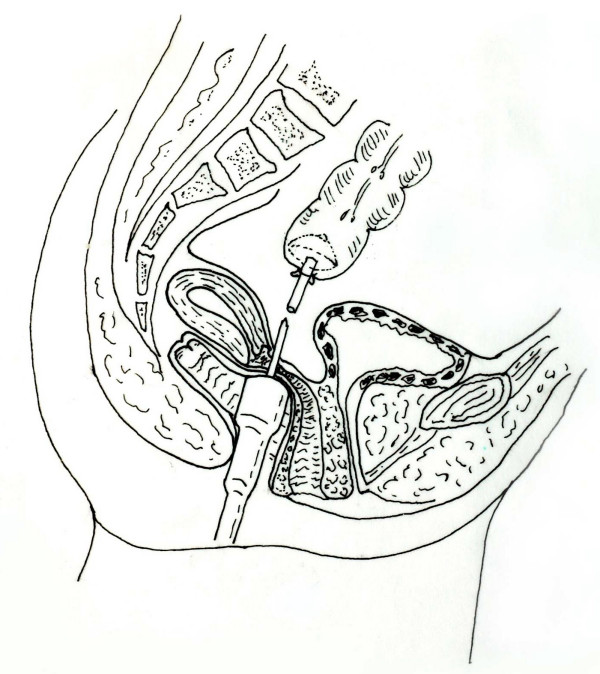
The stapler body was normally introduced into the rectum by the surgeon but inadvertently directed to the anterior rectal and posterior vaginal wall, pushed forward to the anterior fornix of vagina and then meeting with the anvil of the stapler. It lead to an involvement of both the anterior and posterior wall of the vagina and the anterior wall of the rectum into the staple line.

This is probably a unique complication in colorectal surgery. Searching from the PubMed we only found two reports in recent years in the English literature [1,2]. These two cases occurred in patients underwent lower rectal surgery with a past history of hysterectomy. The present case is more complicated than and different from the previous two. Inadvertent formation of colovaginal anastomosis was associated with a rectovaginal fistula. Apparently it was caused by the operator's error. We may image that during the performing of anastomosis the stapler body was normally introduced into the rectum by the surgeon but inadvertently directed to the anterior rectal and posterior vaginal wall, pushed forward to the anterior fornix of vagina and then meeting with the anvil of the stapler. As a result it lead to an involvement of both the anterior and posterior wall of the vagina and the anterior wall of the rectum into the staple line (Figure 1). This could explain why patient did not have any abnormality found during her postoperative three-week hospital stay. With gradual and partial separation of the anterior vaginal wall from the posterior the stool passed through both the vagina and rectum. The colonoscopy undertaken in another hospital before her visiting to us revealed normal anastomosis and rectovaginal fistula when the colonoscope passing freely through the colovaginal anastomosis and going ahead to the colon. The blind rectal stump was overlooked by the endoscopist. Preoperative digital examination of the rectum also did not find the blind rectal stump end, which was 10 cm from the anal verge and could not be reached by the finger without anesthesia. Unfortunately, we did not do a preoperative barium enema because rectovaginal fistula was concerned only. A few of clips left in the vagina wall and loss of the appearance of cervix uteri should attract our attention but we misinterepted it. Transvaginal repair of the rectovaginal fistula did not cure the patient but made the situation even worse because the present of an inadvertent colovaginal anastomosis. Indeed, this unique complication was almost beyond of our expectation.

Application of stapling devices is very popular worldwide. It has provided the surgeons with an excellent tool in gastrointestinal surgery, notably savings in time and makes the procedure being doing in the deep pelvis easier than hand-sewn. On the other hand, rapid and reliable technique of stapling are deceptive difficult to master and require extensive experience to develop proficiency [3]. Well cooperative team work is also mandatory. Complications of using the stapler device include perforation or other injuries of the rectum, incomplete cutting of the intestinal ends with difficulty upon withdrawal of the stapler, postoperative hemorrhage, leakage and stenosis of the anastomotic site. Rectovaginal fistula has been reported but not very often [3]. It was reported at a rate of 2.2% by Antonsen *et al *[4]. Rex and Khubchandani [5] performed a survey to determine the incidence of rectovaginal fistula following low anterior resection. A total of 57 patients were collected from the responders to have postoperative rectovaginal fistulae. In 53 of the 57 patients (93%) a circular stapled anastomosis had been performed. Most of the fistulae occurred from the inclusion of the vaginal wall in a low stapled anastomosis. Arbman [6] stated that double stapler technique was associated with rectovaginal fistula observed after low stapled anastomosis.

The incidence of complication related to the use of stapler probably correlated with the experience and skill of the surgeon. The leading cause of rectovaginal fistula is entrapment of the posterior vaginal wall to the staple line due to poor visualization of the operative field in the deep pelvis especially when the lightening in the operation room is unsatisfactory. Proper placing of a good retractor such as the St. Mark retractor is much helpful. During the approximation of the stapler body and the anvil the posterior wall of vagina is tend to be entrapped into the staple line if the assistant failed to retract it out from the rectal stump. To prevent this type of complication caution must be always taken to ensure not involving the posterior vaginal wall to the staple line and carefully check the situation by digital examination of the vagina before firing the circular stapler.

When establishing intestinal continuity after a Hartmann procedure the surgeon must dissect out the rectal stump in the deep pelvis. If the stump is located below the pelvic peritoneum a used stapler or a rigid sigmoidscope should be inserted into the rectal remnant to help the surgeon identifying the end of stump. The peritoneum covered the stump must be dissected out to make the stump end thinner for stapling anastomosis. Otherwise the thick end will make the cutting and anastomosis incomplete. In male patient inclusion of the posterior wall of the bladder into the staple site may be happened if the rectal stump was not dissected clear of the bladder before stapling. The surgeon who previously operated on our patient did not find the right end of rectal stump but pushing the stapler body forward to the anterior cul-de-sac and an iatrogenic colovagino-rectovaginal anastomosis was unfortunately fashioned.

## Conclusion

Inadvertent formation of colovaginal anastomosis associated with a rectovaginal fistula is a rare complication caused by the operator's error. The complication is avoidable by proper intraoperative technique. The present case again highlights the importance of sufficiently separating the rectal stump from the vaginal wall and ensuring that the posterior wall of vagina is away from the staple line before firing the circular stapler when undertaking a lower colorectal anastomosis.

## Conflict of interests

The author(s) declare that they have no competing interests.

## Authors' contributions

ZY composed this case report.

GL participated the operative procedure and was responsible for the care of the patient.
